# Intrathecal dexamethasone-bupivacaine combination with bupivacaine alone in spinal anesthesia for cesarean delivery

**DOI:** 10.22088/cjim.15.3.414

**Published:** 2024-08-01

**Authors:** Ali Nasiri, Seyed Mohammad Abutorabi, Shahryar Sane

**Affiliations:** 1 Department of Anesthesiology, Faculty of Medicine, University of Medical Sciences, Urmia, Iran

**Keywords:** Pain control, Intrathecal dexamethasone, Spinal anesthesia, Bupivacaine

## Abstract

**Background:**

Postoperative pain management can be achieved by adjuvant medications during the analgesia procedure. The study investigated the effect of intrathecal dexamethasone-bupivacaine combination with bupivacaine alone in spinal anesthesia for cesarean delivery.

**Methods:**

This randomized, double-blind clinical examination included 50 females who had previously experienced a cesarean section. The participants were assigned randomly into two categories: the intervention group, received intrathecal bupivacaine-dexamethasone, and the control group, received intrathecal bupivacaine-normal saline. Levels of pain were evaluated using a 10 cm visual analog scale (VAS) at intervals of 30 minutes, 1 hour, 2 hours after the operation. The span of the sensory block and postoperative analgesia were assessed.

**Results:**

The inclusion of intrathecal dexamethasone with bupivacaine resulted in a significant enhancement in the duration of pain relief during the intervention, lasting for an average of 473.4 ± 39.95 minutes (p<0.001). The duration of sensory and motor block analgesia in the intervention group was more than the control group (128.32 ± 7.30 vs. 92.84 ± 7.84) and (155.6±12.34 vs. 126.16±11.89), respectively (*p*<0.001). Pain score on the VAS scale at 30, 60, and 120 minutes was significantly lower in the intervention group (p<0.001). There was no difference in side effects and onset time between the study groups.

**Conclusion:**

The inclusion of intrathecal dexamethasone alongside bupivacaine has demonstrated enhancement in the duration of sensory block during spinal anesthesia. This improvement was observed without any alterations in the time it takes for the anesthesia to take effect and without any adverse effects during the postoperative period.

The global cesarean section (C-section) rate is significantly increasing (1, 2). Anesthesia during a C-section can make the procedure painless and has few negative effects on the mother and infant. A short period to achieve good anesthesia, little hemodynamic alterations, and few side effects are the features of the optimal anesthetic approach (3). The risk of harm to the mother and the newborn can be reduced by selecting a suitable anesthesia method. 

Most surgical procedures, especially C-sections, are performed under spinal anesthesia (SA) (4). In cesarean section surgeries, two distinct anesthesia techniques are employed; general anesthesia (GA) and regional anesthesia (RA). The utilization of regional anesthesia provides an array of advantages, including prompt restoration of postoperative gastrointestinal functions, improved postoperative pain relief, early mobility for patients during the postoperative period, early bonding opportunities between the mother and baby, as well as reduced risks of medication toxicity for both mother and baby (5).

The most popular local anesthetic drug for cesarean deliveries is bupivacaine in spinal anesthetics (6). Adjuvant drugs are also necessary to reduce the adverse effects of neuraxial analgesics, such as maternal hypotension, shivering, vomiting or nausea, and a faint feeling (7). Dexamethasone, a systemic glucocorticoid, enhances the postoperative recovery process through its effectiveness in alleviating pain, reducing instances of nausea and vomiting (8). When dexamethasone was added to peripheral nerve blocks, postoperative anesthetic action, and blockade duration were prolonged (9). Recent research has revealed that intrathecal dexamethasone had no side effects (10, 11). 

Many studies reported that dexamethasone might be a potential anesthetic adjuvant that can enhance the anesthetic effects of local anesthesia (10, 12, 13). However, little research has been focused on using dexamethasone with a combination of other drugs in C-sections. To fill this gap, this study's objective is to compare how effective intrathecal bupivacaine alone is versus a combination of bupivacaine with dexamethasone in terms of the length of spinal pain relief.

## Methods

This randomized, double-blind clinical trial was approved by the Ethics Committee of Urmia University of Medical Sciences with code: IR.UMSU.REC.1397.483. Moreover, it was approved by the Iranian Registry of Clinical Trials with code: IRCT20221126056613N2. 

This research comprised 50 females aged between 21 to 40 years, all of whom had previously undergone a cesarean section. The patients who were scheduled to undergo a cesarean section procedure with spinal anesthesia were randomly assigned to one of two groups: the intervention group, which received intrathecal bupivacaine-dexamethasone, and the control group, which received intrathecal bupivacaine-normal saline. Prior to their involvement in the study, all participants were provided with comprehensive information regarding all elements of the research and subsequently gave their written consent. Inclusion criteria were the absence of pregnancy complications (previa, accreta, preeclampsia, placental abruption, etc.), stable vital signs, second-to-fifth childbirth, and lack of depression or proven mental disorder. 

The exclusion criteria were patients with a stillbirth history, the presence of a clear anomaly in the fetus, the occurrence of surgical complications (atony, hysterectomy, insertion of the virgin balloon, bleeding more than one liter, pulmonary embolism, oligori), fever of the mother in the first 24 hours, reaching the level of anesthesia higher than T4, failure of spinal anesthesia technique, being under general anesthesia during cesarean section or in the first 24 hours, severe spinal complications (apnea or hemodynamic disorder requiring intubation, cardiac arrest, epidural hematoma). 

After the preparation of the IV line, all patients were adequately hydrated. Premedication was not administered prior to the procedure. Upon the patients' arrival in the operating room, their vital signs were continuously monitored and recorded at 5-minute intervals using non-invasive electrocardiography (ECG), peripheral oxygen saturation (SPO2), and non-invasive arterial blood pressure (NIBP) measurements. Vital signs were subsequently recorded every 15 minutes in the Post Anesthesia Care Unit (PACU). Sedation was administered at the discretion of the anesthesiologist, specifically midazolam at a dosage of 0.025 to 0.05 mg/kg intravenously either before or immediately after the spinal procedure. Patients' demographic data were studied for the two groups and entered into a checklist. Each patient's checklist was marked with a special number, which was the same as the number on the random number table, and was recorded by a person other than the spinal surgeon and the post-cesarean examiner.

Spinal anesthesia was administered using a 25-gauge Quincke spinal needle through a midline approach at the L4-L5 level, with the patients in the sitting position. The control group received a dose of 12 mg of 0.5% hyperbaric bupivacaine mixed with 2 ml of preservative-free normal saline solution. The intervention group received the same dose of bupivacaine, but also received an additional 8 mg of preservative-free dexamethasone under the brand name Dexadic, making the total intrathecal volume 5 ml. After the spinal injection, the patients were placed in a supine position.

 An observer, who was masked to the procedure and not directly involved, assessed sensory and motor blocks every minute for 5 minutes or until the desired block levels of T4 and T10 were achieved. Subsequently, the assessments were made every 15 minutes during the surgery and post-operatively, until the sensory block regressed to the L1 dermatome. The sensory level of the block was determined by assessing the loss of sensation to pinprick, with the forearm serving as a reference point and assessments conducted in a caudal to cephalad direction. The motor block was assessed using the Modified Bromage scale. Readiness for surgery was defined as the loss of sensation to pinprick at the T10 level. Evaluation of the motor block during surgery was suspended until the procedure was completed.

The occurrence of significant low blood pressure (defined as a decrease in the systolic arterial blood pressure by 30% from initial values) was managed by administering an intravenous injection of mephentermine at a dose of 6 mg. Clinically significant slow heart rate (defined as a heart rate below 50 beats per minute) was treated with an intravenous injection of atropine at a dose of 0.6 mg. The total amount of any of these medications or sedatives given was recorded. The length of time that the sensory and motor block lasted, as well as any adverse effects or complications (such as nausea, vomiting, low blood pressure, slow heart rate, etc.), were recorded along with the total dose of additional agents. Pain assessment during surgery or in the post-anesthesia care unit (PACU) was performed using the visual analog pain scale (VAS), which ranges from 0 to 10 (where 0 indicates no pain and 10 represents the most severe pain), every 30 minutes after four regressions of the dermatome block. If the VAS score after the surgery was higher than 6, it was managed by administering an intravenous injection of morphine at a dose of 2 mg. The patients were observed upon discharge from the hospital and again after one month, during which they were asked about any neurological deficits they might have experienced. 

**Statistical analysis: **The continuous variables were assessed through an analysis of variance t-test, and normality was evaluated using Shapiro-Wilk tests. Onset time, sensory block duration, and duration of analgesia were analyzed using appropriate t-tests, and the resulting p-values were reported with a 95% confidence interval. The comparison of categorical variables such as sex, nausea/vomiting, hypotension, bradycardia, ephedrine, and atropine was conducted using a chi-squared or Fisher's exact test. Sensory level was compared using the Mann-Whitney test. A significance level of p<0.05 was chosen. The sample size calculation was based on the study by Akayya et al. (14) considering a 95% confidence interval and a test power of 95%. According to the following formula, 25 individuals were determined for each group.

## Results

In this research study, a total of 50 pregnant females were identified as potential candidates for cesarean section. The results did not indicate any significant statistical distinctions in relation to various factors including age, body mass index (BMI), gestational age, educational attainment, heart rate (HR), systolic blood pressure (SBP), diastolic blood pressure (DBP), and peripheral capillary oxygen saturation (SpO2). The detailed information is provided in table 1.

**Table 1:** Demographic and clinical characteristics of the two studied groups

**Variable**	**Intervention group** **(n=25)**	**Control group** **(n=25)**	**P-value**
**Age (year)**	26.6 ± 4. 13	27.16 ± 4.39	0.645
**BMI (kg/m2)**	28.43 ± 1.07	28.98 ± 1.45	0.138
**Gestational age (week)**	39.0±0.9	38.8±0.8	0.366
**Educational level (year)**	8.28 ± 4. 43	8.12 ± 4.53	0.935
**HR (b/min)**	86.48 ± 6.30	90.88 ± 5.73	0.013
**SBP (mmHg)**	118.32 ± 9.61	117.68 ± 8.75	0.708
**DBP (mmHg**	67 ± 3.24	66.12 ± 3.84	0.386
**SpO2 (%)**	97.6 ± 0.95	97.4 ± 0.96	0.46

Body mass index (BMI), Heart rate (HR), Systolic blood pressure (SBP), Diastolic blood pressure (DBP), and Peripheral capillary oxygen saturation (SpO2).

However, statistical analysis revealed no significant difference between the two groups (P=0.758) (P=0.596). The duration for the highest level of motor block was 5.8±1.15 minutes in the intervention group and 6.08±1.8 minutes in the control group (P=0.668). With the Modiﬁed Bromage scale, the mean anesthesia time for the motor block was 2.88±0.33 minutes in the intervention group and 2.84±0.37 minutes in the control group (P=0.687). 

Overall, there was no significant variation observed in these variables between the two groups (table 2). Table 3 indicates that there is no statistically significant distinction among the groups under examination in terms of pain score at minute 0 (P=0.429). Nevertheless, the intervention group exhibited significantly lower pain scores at minutes 30, 60, and 120 (1.1±0.6, 1.4±0.5, 1.9±0.6, respectively) compared to the control group. Hence, there exists a statistically significant difference between the two groups during these time intervals. In regard to side effects, the control group experienced a higher occurrence among patients (shivering: 11(44%), vomiting: 4(16%)). Nonetheless, no notable difference was observed between these groups according to table 4.

**Table 2:** Characteristics of the analgesic profile, sensory and motor block between the two studied groups

**Variables**	**Intervention Group** **(n=25)**	**Control Group** **(n=25)**	**P-value**
**Duration of analgesia (min)**	473.4 ± 39.95	204.08 ± 19.38	<0.001*
**Meantime of anesthesia for sensory block**	128.32 ± 7.30	92.84 ± 7.84	<0.001*
**Meantime of anesthesia for the motor block**	155.6±12.34	126.16±11.89	<0.001*
**meantime of anesthesia reached the levels of T10**	1.64 ± 0.75	1.76 ± 0.93	0.758
**meantime of anesthesia reached the levels of T4**	4.72 ± 0.93	4/92 ± 1/35	0. 596
**meantime of anesthesia reached the highest level of motor block**	5.8±1.15	6.08±1.8	0.668
**meantime of anesthesia of the motor block (Modiﬁed Bromage scale)**	2.88±0.33	2.84±0.37	0.687

**Table 3:** Pain perception (VAS score) among the control and intervention group

**Time**	**Measures**	**Intervention Group** **(n=25)**	**Control Group** **(n=25)**	**P-value**
**Minute-0**	**Mean±SD**	0.3±0.4	0.3±0.6	0.429
	**range**	0.0–1.0	0.0–2.0	
**Minute-30**	**Mean±SD**	1.1±0.6	1.4±0.5	0.008*
	**range**	0.0–2.0	0.0–2.0	
**Minute-60**	**Mean±SD**	1.4±0.5	1.9±0.6	<0.001*
	**range**	1.0–2.0	1.0–3.0	
**Minute-120**	**Mean±SD**	1.9±0.6	2.5±0.7	<0.001*
	**range**	1.0–3.0		

**Table 4:** Side effects among the studied groups

**Side effects**	**Intervention Group (n=25)**	**Control Group (n=25)**	**P-value**
**Nausea**	4(16%)	5(20%)	1
**vomiting**	2(8%)	4(16%)	0.667
**Shivering**	7(28%)	11(44%)	0.337
**Headache/ dyspnea**	0(0%)	0(0%)	-
**Bradycardia**	4(16%)	5(20%)	1
**Hypotension**	4(16%)	5(20%)	1

## Discussion

One of the main concerns of anesthesiologists was the duration of anesthesia and developing methods to manage it. Consequently, this study revealed that adding dexamethasone to bupivacaine improved motor and sensory block and prolonged analgesia in spinal anesthesia. But the groups did not show a difference in the onset time of sensory and motor block and postoperative complications. 

The findings of this study corroborate previous research conducted on a cohort of 50 patients who were scheduled for orthopedic surgery with spinal anesthesia. The results indicate that the duration of sensory block in the case group was significantly longer than in the control group (15). Similarly, a separate investigation demonstrated that the concurrent administration of dexamethasone and bupivacaine during spinal anesthesia led to a substantial increase in the length of sensory and motor block, as well as surgical analgesia and postoperative pain-free period, without any adverse effects (16). In yet another study, three groups of male patients scheduled for transurethral prostatectomy under spinal anesthesia were administered hyperbaric bupivacaine with dexamethasone, meperidine, or normal saline. The dexamethasone group exhibited superior sensory and motor block characteristics, and their postoperative analgesic duration was comparable to the control group (p <0.001). Compared to the control group, both the dexamethasone and meperidine groups displayed more favorable sensory and motor block features and postoperative analgesic duration. However, patients in the meperidine group experienced increased sedation and pruritus. These factors, along with the higher satisfaction scores reported by patients in the dexamethasone group, serve to reinforce the benefits associated with dexamethasone. Moreover, patients in the dexamethasone group experienced fewer hemodynamic events and no sedation (17).

In another research study, the combination of dexamethasone and bupivacaine was utilized to effectively prolong the duration of pain relief in patients who received an ultrasound-guided interscalene brachial plexus block. The amount of opioids required, as measured using the equivalent dosage of oxycodone, was significantly lower in the group that received dexamethasone compared to the control group during the initial 24-hour period, and remained similar thereafter (18). These findings differ from a previous study conducted by Akayya et al. (14), where the addition of 4 mg of dexamethasone to bupivacaine in patients undergoing inguinal herniorrhaphy under spinal anesthesia did not result in an extended time until the first request for analgesics, and only provided minimal analgesic effects 12 hours post-surgery. It is important to note that the variation in these outcomes could be attributed to differences in the administration routes, dosages, and timing of dexamethasone.

The results of this study demonstrated that the introduction of intrathecal dexamethasone alongside bupivacaine does not result in a different onset time for sensory and motor block compared to the administration of bupivacaine alone during spinal anesthesia for cesarean section. Similarly, another study found that intrathecal administration of dexamethasone in combination with levobupivacaine does not alter the onset time of sensory block when compared to the administration of levobupivacaine alone (19). However, contrasting findings were reported by Knezevic et al., who observed that perineural administration of dexamethasone extended the onset time of both sensory and motor block and resulted in the earlier occurrence of pain than anticipated (20).

In our study, we observed that while there were no significant distinctions in side effects between the two groups, individuals who did not receive dexamethasone exhibited a higher incidence of these effects. This observation suggests that following systemic absorption, the administration of 8 mg dexamethasone influences the physiological functions of organs by directly affecting the solitary tract nucleus, serotonin neurotransmitter interaction, tachykinin proteins NK1 and NK2, and alpha adrenaline (21).

In this scholarly investigation, bupivacaine was selected as the subject of examination due to its decreased occurrence of cardiovascular and neurologic adverse reactions, as well as fewer instances of motor block in comparison to bupivacaine (22). Dexamethasone was utilized as an additional substance owing to its effectiveness, safety, and economical nature. Certain researchers argue that this practice is correlated with the anti-inflammatory properties of dexamethasone, which hinder the generation of inflammatory molecules like prostaglandins and glutamate within the spinal cord (23). The significant prolongation of this study's sensory and motor block can be due to the vasoconstrictive qualities of steroids. It has been proven that steroids increase vascular tone and so-called vasoconstriction by various mechanisms, including cytosolic glucocorticoid receptors of vascular smooth muscle cells, α-adrenergic, and angiotensin II receptors (24). On the other hand, vasoconstriction in the subarachnoid space lengthens the time that intrathecal medications have an effect during spinal anesthesia (25).

Certain limitations were encountered in our investigation. We did not evaluate the relationship between dose and response of dexamethasone. Furthermore, the measurement of pain intensity proved to be arduous, particularly for individuals experiencing intense and long-lasting discomfort. An inherent restriction of the visual analog scale (VAS) is the ceiling effect, wherein patients are unable to accurately quantify their escalating pain. Further research is imperative to appraise varying doses of transiently effective steroids in regard to postoperative pain upon completion of surgery.

Through this research, it has been demonstrated that the inclusion of intrathecal dexamethasone alongside bupivacaine has led to notable enhancements in both the sensory and motor block duration during spinal anesthesia. No alterations were observed in terms of the onset time or complications. Thus, further exploration is warranted to establish a comprehensive framework for incorporating intrathecal dexamethasone into local anesthetics during spinal anesthesia, with the ultimate goal of improving the duration of anesthesia in cesarean deliveries.
